# Book Review

**Published:** 1900-06

**Authors:** 


**Cancer.** By W. ROGER WILLIAMS. Pp. 189—396. **Sarcoma.** By W. ROGER WILLIAMS. Pp. 485—554. New York: William Wood and Company. 1898. Mr. Roger Williams has long been known for his patient research on the questions which are dealt with in these articles reprinted from vol. xvii. of the *Twentieth Century Practice of Medicine.* They deal with the statistical and pathological, rather than the clinical and surgical aspects of their respective subjects. Both works show a wide acquaintance with the literature of these diseases, and we do not know any more able and exhaustive treatises. The correlation of cancer and phthisis has been carefully worked out, and the author gives good reasons for his belief that most cancer patients are the surviving members of tuberculous families. The evidence pointing to the existence of a specific cancer microbe is regarded as altogether inconclusive. Reprints of such comprehensive articles deserve indexes.

**Annual Report of the Smithsonian Institution, 1897.** Washington: Government Printing Office. 1898.—The Royal Society is the nearest approach to anything we have in England to correspond to the Smithsonian Institution in America. The latter, however, is subsidised by the Government of the United States. The present volume is the annual report of the Institution for the year ending June 30th, 1897. The secretary's letter, presenting the report to Congress, at the commencement of the volume, is dated April 14th, 1898, nearly ten months after the end of the year, consequently some of the papers in the general appendix seem somewhat out of date. Presumably this delay in publishing is unavoidable; but it seems somewhat of a pity that such a valuable collection of papers as are here gathered together should not be given to the world sooner. After the secretary's report of the proceedings of the Institution comes what is called the general appendix, the object of which—according to the introduction—is "to furnish brief accounts of scientific discovery in particular directions." It consists of papers by scientists from all parts of the world. The only two papers that can at all be called medical in character are the masterly address of Sir M. Foster, delivered before the British Association at Toronto, on "Recent Progress in Physiology," and an account of mescal intoxication by Havelock Ellis. Amongst the other papers of special interest are the lecture by Sir W. Crookes on "Diamonds," the essay by Abbott H. Thayer on "The Law which Underlies Protective Coloration," and "Letters from the Andrée Party;" but there is not a single paper in the volume which will not repay careful perusal.

**Bibliographia Medica [Index Medicus].** Tome I., No. 1. Janvier, 1900. Paris: Institut de Bibliographie.—In its most earnest words the medical world should express its thankfulness for this publication, which is intended to take the place of the defunct *Index Medicus.* Those who had learned to value that compilation will be more than glad to have this excellent substitute, formed almost exactly on the old lines for the credit of which so much is due to the energetic and scientific labours of American bibliographers. We trust that M. Baudouin *et ses confrères* will meet with the support they deserve. The one thing that is lacking in this new publication is the consideration of the literature issued between the last record of the *Index Medicus* and the first number of its successor. But doubtless provision is being made for the supply of this at an early date. There must be no break in the indispensable work of the two publications.

**Transactions of the Jenner Institute of Preventive Medicine** (late *British* Institute of Preventive Medicine). Second Series. London: Macmillan & Company, Limited. 1899.—Through the munificence of Lord Iveagh, the Jenner Institute is now housed and endowed on a scale commensurate with the importance of the work undertaken. Amongst the records of the work of the year are papers upon the structure of bacteria, on the thermophilic and photogenic properties, and on the uses of bacteria in technical trade processes. Dr. Salter's paper on the pathogenicity of the pseudo-diphtheria bacillus and its relation to the Klebs-Loeffler organism is another link in the chain of evidence going to prove that the pseudo-organism is only a modified form of the virulent bacillus, and that the two varieties are readily convertible into one another. This being the case, the value of Neisser's diagnostic stain, dealt with by Dr. R. T. Hewlett, is not so great as was at first supposed, for Hoffmann's bacillus, whlch does not take on the characteristic staining reaction, may subsequently become as dangerous as the typical diphtheritic organism. We have not ourselves found Neisser's test altogether trustworthy, many of the organisms ordinarily found in the mouth giving the characteristic granular staining. Valuable hints will be found in the "Laboratory Notes" on the staining of flagella and capsules, and on the methods of examining specimens for anthrax. It has been found in the laboratory that 25 per cent. of diphtheria cultures on blood-serum yield positive results within six or seven hours, a point of great practical importance in a disease in which early diagnosis is of the greatest importance. In a note on the commonly-reported failure to find the amœba coli in cases of tropical abscess of the liver, the direction is given to test when the walls of the abscess cavity begin to break down after drainage, for it is then that the amœba makes its appearance in the discharge. The *Transactions* are a valuable addition to bacteriological literature, and the profession is to be congratulated on the increased facilities for research afforded by the Institute.

**The Thermal Waters of Bath.** By Gilbert A. Bannatyne, M.D. Pp. 87. Bristol: John Wright & Co. 1899.—This little book by Dr. Bannatyne is written with a single eye—it is intended for doctors. It explains the methods of treatment used in the bathing establishments in a thorough workmanlike fashion; it gives a list of what diseases may be cured or benefited by such treatment, and that, too, without describing the diseases; and it summarises the results which have been obtained at the Mineral Water Hospital in each disease. The book is carefully and scientifically written, and will be of great service to the medical profession.

**The Diseases and Primary Tumours of the Thymus Gland.** By H. D. Rolleston, M.D. Pp. 23. London: The Medical Publishing Company, Limited. [n.d.]—This essay is worthy of a better garb than a small undated pamphlet. It opens up a comparatively new field, and is of considerable importance in connection with the modern treatment of myxœdema and exophthalmic goitre. The author gives evidence in favour of the view that many of the morbid growths arising in the anterior mediastinum are due to the presence there of the remains of the thymus.

**The Sanitation of British Troops in India.** By E. Carrick Freeman, Captain R.A.M.C. Pp. 106. London: The Rebman Publishing Company (Ltd.). 1899.—We can with confidence recommend this book to the notice of young medical officers going to India. It gives in a small compass the various dangers to health to which the British soldiers serving in that country are exposed, and the sanitary precautions that should be taken to maintain them in a high state of efficiency. The author clearly shows how diseases of the most serious nature may be disseminated through the gross ignorance of the native sweepers. The recommendations for the purification of a suspicious water are sound and practical; and as the value of the Pasteur-Chamberland filter is fully established, its universal adoption in India is strongly recommended. Strict and capable supervision of all cooking arrangements is rightly advocated, and some excellent advice is given as to the precautions that should be taken to prevent the pollution of milk. The housing of troops in the tropics is of the first importance, and in reference to this there is much valuable information.

**The Treatment of Lateral Curvature of the Spine.** By Bernard Roth. Second Edition. Pp. viii., 141. London: H. K. Lewis. 1899.—The author of this work is well known as an advocate of the treatment of lateral curvature of the spine by "posture and exercise," and not by the so-called "spinal supports." There can be no doubt that he is quite right, and all modern surgeons will agree in the wish "that the publication of this little book will do something to check the unscientific and often disastrous treatment of lateral curvature of the spine by spinal supports and prolonged rest." An appendix containing a series of one thousand consecutive cases of lateral curvature treated by the author is given at the end of the book.

**A Code of Rules for the Prevention of Infectious and Contagious Diseases in Schools.** Fourth Edition. Pp. 47. London: J. & A. Churchill. 1899.—The rules contained in this code, issued by The Medical Officers of Schools Association, are quite excellent, and their careful enforcement in all schools should reduce the danger of the introduction or spread of infectious disease to a minimum.

**Clinical Lectures on Neurasthenia.** By Thomas D. Savill, M.D. Pp. xv., 144. London: Henry J. Glaisher. 1899.—The etiology, diagnosis, pathology, and treatment of neurasthenia are fully discussed in this volume, which consists of five lectures delivered in 1891, 1897, 1898. The disease is shortly defined as an irritable weakness of the nervous system, brought about by toxic blood states, malnutrition, fatigue, or emotional shock. The writing is lucid, and numerous cases are cited in support of the author's conclusions. The chief defect is that there is some amount of repetition, which, though no doubt useful in lectures, is quite unnecessary and irritating in a book. There is a good index, and a very complete bibliography is appended.

**Constipation and its Modern Treatment.** By George Herschell, M.D. Second Edition. Pp. 91. London: Henry J. Glaisher. 1899.—This book deals with the treatment of obstinate or confirmed cases of constipation by other methods than the routine administration of drugs. It is well known that the drug treatment of chronic constipation may end in failure, larger and larger doses of medicine being required. The practitioner will find some useful advice here as to its treatment by douches, electricity, exercises, and by a method which is still unfamiliar, and of which further experience is necessary before an opinion can be pronounced on its merits—namely, vibration applied to the abdominal viscera by a special apparatus. We recommend the book, as it contains many useful suggestions, and the directions are plain and marked by common sense.

**The Pocket Case Book for District and Private Nurses.** London: The Scientific Press, Limited. [N.D.].—Nurses will probably find this little book useful in which to note down details of cases, which are apt to escape the memory, and on which they have to report to the doctor in charge. It can be conveniently carried in the pocket, and will tend to increase of accuracy and method in observation on the nurse's part. The headings under which the notes are to be made are well selected.

**Open-Air Treatment of Consumption—Seven Years' Experience in England.** By JANE H WALKER, M.D. Pp. 15. London: J. & A. Churchill. 1899.—This reprint from the *Practitioner* contains a summary of seventy-eight cases, and the general result of these is said to be "encouraging, and presents features of hopefulness, even in advanced phthisis, which a few years ago would have seemed quite beyond the bounds of possibility."

**Growing Children: their Clothes—and Deformity.** By E. NOBLE SMITH. Pp. 23. London: Smith, Elder, & Co. 1899. —Contained in this small pamphlet are a few remarks on children's clothes, etc., most, if not all, having long been common knowledge.

**Organothérapie ou Opothérapie.** Par le Dr. C. HILLEMAND. Pp. 53. Paris: G. Steinheil. 1899.—The author has given us a useful review of the employment of animal extracts in medicine and of the results attained. Such great hopes were entertained when the first successes of this method were reported, that extracts have been prepared from almost every organ of the body. After analysing these attempts, Hillemand finds that only one, the thyroid treatment for myxœdema, has been established beyond all doubt, but there is a good deal of evidence in favour of several others, such as the supra-renal. We must congratulate the author on his clear and able discussion of a subject on which many exaggerated statements have been made.

**Hygiene and Public Health.** By B. ARTHUR WHITELEGGE, M.D. [Eighth Edition.] Pp. viii., 588. London: Cassell and Company, Limited. 1899.—This excellent manual has been previously noticed with approval in these pages. In the present edition the author has incorporated in the text some account of the more recent developments in many branches of hygiene. In particular, a summary of the important changes in administration introduced by the Vaccination Act of 1898 is given in chapter xii. This book is certainly one of the most concise and helpful of the smaller text-books on Public Health.

**Lessons on Prescriptions and the Art of Prescribing.** By W. Handsel Griffiths, Ph.D. [Fourth] Edition, adapted to the British Pharmacopœia of 1898. Pp. x., 148. London: Macmillan and Co., Limited. 1899.—This useful little book should be in the hands of every medical student, inasmuch as it teaches what a large proportion never learn.

**Merck's Manual of the Materia Medica.** Pp. vi., 236. Darmstadt (Germany): E. Merck. 1899.—Part i. affords at a glance a descriptive survey, in one alphabetical series, of the entire Materia Medica of to-day. Part ii. contains a summary of therapeutic indications for the employment of remedies, arranged according to the pathological conditions to be combated. Part iii. presents a classification of medicaments in accordance with their physiological actions. The manual is designed to meet a need which every practitioner has often experienced, when the memory is treacherous. The publisher has laboured long and earnestly to render this little volume "a firm and faithful help to the practitioner in his daily round of duty."

**Transactions of the Epidemiological Society of London.** Vols. XVII., XVIII. London: Shaw & Sons. 1898-99.—These volumes contain much of prime importance to the student of public health. No less than five papers are concerned with the epidemic history of enteric fever, as exemplified in the experience of Munich, Clifton, Norwich, Nottingham, and the Netherlands. In vol. xviii. Dr. Ransome contributes a valuable paper on "The Prospect of Abolishing Tuberculosis," which is of much interest at the present time.

**Reports and Papers of the American Public Health Association.** Vol. XXIII. Concord: The Rumford Press. 1898. Vol. XXIV. Columbus: The Berlin Printing Company. 1898.—These two handsome volumes contain a large amount of interesting and valuable material, presented at the twenty-fifth and twenty-sixth annual meetings of the Association. Two papers of especial interest in the 1897 volume come from the laboratories of the Minnesota and Philadelphia Boards of Health, on the Diagnosis of Typhoid Fever by the Blood reaction, generally known as Widal's. In the volume for 1898 we note two papers on leprosy, a disease still maintaining a few foci in America, to which country the recent annexation of the Sandwich Islands forms an added danger, giving rise to the question, "Ought we to reopen the Leper Asylums?" The Etiology of Yellow Fever is also fully considered; and a paper on Anthrax in relation to tanneries points out a source of considerable risk to workers in certain trades to which all countries are exposed, but which is generally not fully recognised.

**Transactions of the Medical Society of London.** Vol. XXII. London: Harrison and Sons. 1899.—The work done by the Society during its one hundred and twenty-sixth session, under the genial presidency of Mr. Edmund Owen, commences with the usual discourse, where the hope was expressed that "the spirit of tolerance and fraternity which Lettsom breathed into that little group of medical friends in the month of May, 1773, might continue to increase among our Fellows, not only for the session which is now beginning, but until time itself shall be no more." Dr. Allchin gives some considerations preliminary to the study of dyspepsia: he points out other considerations more recent than the historic turtle of June, 1775, when Lettsom feasted the young Society. Sir William Broadbent describes the conduct of the heart in the face of difficulties, and Dr. Robert Maguire endeavours to explain cases of death from functional nervous disease as being due to exhaustion of grey matter. The Lettsomian lectures on "Some Clinical Aspects of Granular Kidney," by Dr. Samuel West, occupy a considerable portion of the volume, and the annual oration by Alban Doran on "Shakespeare and the Medical Society" opened up a mine which is never exhausted.

**Medico-Chirurgical Transactions.** Vol. LXXXII. London: Longmans, Green and Co. 1899.—Together with the presidential address by Mr. Thomas Bryant, we have in this volume a number of very valuable papers read before the society during the past year and the annual financial statement, which shows that the society is in a very flourishing condition. The papers are fully reported and are, many of them, well illustrated, and will amply repay a careful perusal.

**The Diagnosis and Treatment of Syphilis.** By Tom Robinson, M.D. Second Edition. Pp. 138. London: J. & A. Churchill. 1900.—In 1887 we reviewed the first edition of this work, and as in the present edition no substantial alterations have been made, and no new subject discussed, extended comment on our part is unnecessary. We are told in the preface, where a curious and to us unintelligible distinction is drawn between the need and necessity of altering the work, that some corrections have been made. If the necessity for the production of a third edition should ever arise, we hope that the errors still remaining will receive attention. On p. 81 the word "thrombolic" occurs; on p. 85 "joint should be "joints"; on p. 112 "metacarpophalangeal" should be "metatarsophalangeal," and on p. 19 the definite article is left out before the word "vesicle." The expressions "occurs for," on p. 66, and "settled down and commenced," on p. 85, are, to say the least, inelegant. Again, the phrases "destroys fissures," on p. 106, "oxygen . . . is destroyed," on p. 118, and "process of slipping off the periosteum," on p. 107, are objectionable. The second sentence on p. 112 requires correction. We do not feel that this small treatise on syphilis has any special excellence which can justify us in recommending it strongly to students or practitioners.

